# 

**Published:** 1918-03-09

**Authors:** 


### Manager's Notices.

**Now Ready, "The Hospital" Index to Vol. LXII., April 1917 to Sept. 1917. Price 2½d. per copy post free.**

**All letters on business matters must be addressed to The Manager, "The Hospital," 28 and 29 Southampton Street, London, W.C. 2.**

Rates of Subscription (payable in advance).

| | | |
|---|---|---|
| UNITED KINGDOM. | | |
| Three Months | (including Postage) | 2s. 0d. |
| Six Months | do. | 4s. 0d. |
| Twelve Months | do. | 6s. 6d. |
| FOREIGN AND COLONIAL. | | |
| Three Months | (including Postage) | 2s. 6d. |
| Six Months | do. | 5s. 0d. |
| Twelve Months | do. | 8s. 8d. |

**Special Rates** will be quoted to those institutions that agree to send all their public notices to The Hospital, so as to make it a file of such announcements for ready reference.

**Subscriptions,** which may commence at any time, are payable in advance. Cheques and Post-Office Orders should be crossed London County and Westminster Bank, Covent Garden Branch, and made payable to the Manager of The Hospital.

**It is especially requested that remittances be made by Cheque or Postal Order, and in halfpenny stamps only when the amount is under sixpence.**

### Disabled Men as Limb-makers.

Mr. Hodge has stated that the Ministry of Pensions is continuously seeking to train disabled men as limb-makers. The present difficulty is not so much one of providing beds as of finding limb-makers who can turn out limbs of approved pattern, and of securing the services of surgeons who are specialists in limb-work. In the present state of the limb industry it would probably be more expensive to establish new centres in Wales than to send the men to the existing centre in Cardiff.

### Excess Profits and Donations to Hospitals.

Mr. Bonar Law, in answer to a question put by Sir Samuel Roberts in the House of Commons, said that general donations to hospitals and for other charitable purposes are not admissible deductions in computing liability to income tax and excess profits duty. Such legal recognition would involve the consequence that the larger proportion thereof would in effect be a contribution by the State, and, the Chancellor of the Exchequer added, it is most desirable that grants by the State should be made in a direct form.

### THE LONDON HOSPITAL.

### King's College Hospital.

THE annual Court of Governors of this institution was held at the hospital, Denmark Hill, on Thursday, February 28, with Viscount Hambleden in the chair. The annual report stated the average number of beds in daily use to be 565, of which 144 were occupied by civilian and 421 by military patients (255 officers). The financial condition of the hospital continued to be a cause of the greatest anxiety to the committee. The total expenditure for the year was £80,795, showing an increase of 10 per cent. on that for 1916. After deducting £40,608 received for military patients, £40,187 had to be provided from the hospital funds, £5,000 more than in the previous year. The income from ordinary sources was £23,282, so that the deficiency of income was nearly £17,000. The increase in the expenditure had been mainly in provisions (£2,500), coal and fuel oil (£1,700), and salaries and wages (£2,200). The committee reported that the War Office had gone into occupation of "The Platanes," Champion Hill, which was used for housing the medical students, but up to the present the War Office had made no arrangements to pay the sums for ground rent and insurance which were formerly paid by the occupants of the hostel.

### THE SPECIAL APPEAL.

As it was obvious that the hospital would have to rely on increased subscriptions and donations to make up the deficiency in its annual income, there had been instituted a Special Appeal Committee with the object of raising, by systematic effort, a sum sufficient to meet the deficiency in ordinary income, and, if possible, to reduce the debt to the bank, which still remained at nearly £60,000, of which £9,000 was on account of the medical school. Lady Hambleden had, in addition, founded a Ladies' Association towards the same end. During the year this organisation had handed to the hospital over £2,000.

### Annual Meetings.

BOOKSELLERS' PROVIDENT INSTITUTION.—Stationers' Hall, Wednesday, March 13, 1918, at 7 P.M. The meeting will be followed by a concert. The Right Honourable Augustine Birrell, P.C., M.P., will deliver an address.

CANCER HOSPITAL (FREE), FULHAM ROAD, S.W.—Wednesday, March 13, 1918, at 4 P.M., the Right Hon. the Earl of Northbrook, President, in the chair.

HOME HOSPITALS ASSOCIATION.—Fitzroy House, Fitzroy Square, W., Friday, March 15, at 5 P.M., Sir John Bland Sutton, F.R.C.S., in the chair.

NATIONAL HOSPITAL FOR THE RELIEF AND CURE OF THE PARALYSED AND EPILEPTIC (ALBANY MEMORIAL).—The Hospital, Queen Square, W.C., Tuesday, March 26, at 4.30 P.M.

SEAMEN'S HOSPITAL SOCIETY.—Prince's Restaurant, Piccadilly, Thursday, March 14, at 3 P.M., Sir Rosslyn Wemyss, K.C.B., First Sea Lord of the Admiralty, in the chair.

### Matrons' and Sisters' Appointments.

BOROUGH HOSPITAL, BIRKENHEAD.—Miss E. S. Cooper has been appointed night sister. She was trained at the Royal Infirmary, Oldham, and has been sister at the Infirmary, Stockport.

CHILDREN'S HOSPITAL, BIRKENHEAD.—Miss A. Watson has been appointed sister. She was trained at Bootle Borough Hospital, Liverpool, and has been staff nurse at Highfield Military Hospital, Knotty Ash, Liverpool.

CITY HOSPITAL, GRAFTON STREET, LIVERPOOL.—Miss Mary B. Gallagher has been appointed sister. She was trained at Belfast Infirmary and Purdysburn Fever Hospital.

COUNTY AND CITY ROYAL INFIRMARY, PERTH.—Miss Emily Godfrey has been appointed night sister. She was trained at the County Hospital, York, later being sister there.

DRINKWATER PARK HOSPITAL, PRESTWICH, NEAR MANCHESTER. —Miss Ruth Sterritt has been appointed sister. She was trained at Rotherham Hospital and Dispensary and at St. Helen's Sanatorium, and has been sister at Kendray Fever Hospital, Barnsley. She has also done private nursing.

GENERAL HOSPITAL, ALTRINCHAM.—Miss Gladys Longley has been appointed night sister. She was trained at the Infirmary, Stockport, and has been staff nurse at the War Hospital, Chester.

GENERAL INFIRMARY, MACCLESFIELD.—Miss Ethel Nixon has been appointed sister of the women's and children's ward. She was trained at Hull Royal Infirmary.

HODNET HALL AUXILIARY HOSPITAL, HODNET.—Miss Turner has been appointed sister. She was trained at the General Hospital, Birmingham, and has been nursing at Birmingham Orthopædic Hospital.

HOSPITAL ST. CROSS, RUGBY.—Miss G. Draper has been appointed night sister. She was trained at the Taunton and Somerset Hospital, Her Majesty's Hospital, Stepney Causeway, and at the Isolation Hospital, Winchmore Hill, has been night sister at the Taunton and Somerset Hospital, and has also done private nursing.

INFECTIOUS DISEASES HOSPITAL, PORTSMOUTH.—Miss Ada Freeman has been appointed night sister. She was trained at St. Bartholomew's Hospital, London, and at the Western Fever Hospital, Fulham.

LEAF HOMŒOPATHIC COTTAGE HOSPITAL, EASTBOURNE.—Miss Florence Pitt has been appointed matron. She was trained at the Royal Isle of Wight County Hospital, Ryde, and has since held various appointments.

NORTH HERTS AND SOUTH BEDS HOSPITAL, HITCHIN.—Miss Florence Warner has been appointed matron. She was trained at the Prince of Wales' General Hospital, Tottenham, later being assistant matron there, and has been matron of Bridgend Hospital.

OCHIL HILLS SANATORIUM, MILNATHORT.—Miss M. L. Upstone has been appointed sister. She was trained at the Infirmary, Birmingham, has been staff nurse at The Grange Hospital, Southport, and has done private nursing.

PRINCESS ALICE HOSPITAL, EASTBOURNE.—Miss Elizabeth Marchant has been appointed sister. She was trained at Grimsby and District Hospital, and has been sister at the General Hospital, Accrington.

QUEEN ALEXANDRA'S MILITARY NURSING SERVICE FOR INDIA. —Miss Agnes Mary Irwin, Miss Effie Florence Grove, Miss Beatrice Anderson, Miss Kathleen May Roxburgh Carmichael, and Miss May Mildred Edith Low have been appointed nursing sisters.

ROYAL NATIONAL HOSPITAL FOR CONSUMPTION, VENTNOR.— Miss Nellie Gertrude Beeham has been appointed night sister. She was trained at Peterborough General Hospital, Brookfield Hospital, and at Queen Charlotte's Hospital. She has been first assistant nurse at Brookfield Hospital and sister at Queen Charlotte's Hospital and at Bromley Cottage Hospital.

ROYAL SOUTH HANTS AND SOUTHAMPTON HOSPITAL.—Miss M. Hindle has been appointed sister. She was trained at the Royal Infirmary, Huddersfield.

ROYAL SURREY COUNTY HOSPITAL, GUILDFORD. — Miss Dorothy James has been appointed sister. She was trained at Chester Royal Infirmary, has been theatre and ward sister at Wirral Children's Hospital, ward sister at Worcester General Infirmary, and has served under the British Red Cross Society both at home and abroad.

ST. LUKE'S WAR HOSPITAL, HALIFAX.—Miss Amy Moseley has been appointed sister. She was trained at Hull Royal Infirmary, where she was later sister.

SIR FREDERICK MILNER RECUPERATIVE HOSTEL, LONDON, N.W.—Miss B. A. Hope, A.R.R.C., has been appointed matron. She has lately been matron of Thorncombe Military Hospital, Bramley, Surrey, and of the Manor House Hospital, Folkestone.

SURBITON HOSPITAL.—Miss Dora Thompson has been appointed sister. She was trained at Bolton Infirmary and Dispensary.

**NOTE.—Particulars of Appointments for insertion in this column will be welcomed.**

### Gifts and Bequests.

MISS MARY MARGUERITE GEORGINA TAYLOR, of 2 Side Cliff Road, Roker, Sunderland, left £50 to the Hospital for Diseases of the Nose, Throat, and Ear, Rye Hill, Newcastle.

THE Chelsea Hospital for Women has received a donation of £200 from Captain T. Sorby, who was recently a patient in the military section, towards providing an *x*-ray installation.

MRS. HARMON MARMONT, of Crouch Hill, London, W., left £1,000 to the Great Northern Hospital.

MISS CHARLOTTE ELLIS, of the Hall, Belgrave, Leicestershire, who died on September 20 last, left £50 each to the Leicester C.O.S., the Leicester Infirmary, and the Institution of Trained Nurses, Leicester.

LORD BARNARD has sent a gift of grapes to the Royal Hospital for Incurables, Putney Heath.

ESTATE duty has been paid on a provisional sum of one million pounds in connection with the will of Mr. Philip Henry Vaughan, formerly a director of the Bristol Brewery Company. Mr. Vaughan left £5,000 to the Queen Victoria Convalescent Home, Bristol, £500 to the Bristol Royal Infirmary, and £15,000 and property to the Home for Incurables, in trust.

THE "Peter Coats Trust" has sent a donation of £500 to the Eversfield Chest Hospital, St. Leonards-on-Sea, and £100 has been received from the trustees of the late George Crowhurst Rubie, J.P., of Dover.

IN addition to a bequest of £5,000 to King Edward's Hospital Fund for London, Mrs. Lizzie G. Nepomoucky Oumiroff, of Portland Place, W., has left £10,000 for the benefit of disabled or invalided sailors and soldiers.

MRS. SARAH COLEMAN, of Birmingham, bequeathed £1,250 each to the Queen's Hospital, the Ear and Throat Hospital, and the Eye Hospital, at Birmingham.

THE residue of the estate of Miss Sarah Hall, of Moseley, after the death of one legatee, is left to be divided amongst seventeen charities, including the General Institute for the Blind, the Ear and Throat Hospital, the Hospital for Women, and the Eye Hospital, all of Birmingham; and the Cancer Hospital and the National Hospital for the Paralysed and Epileptic, London.

MR. GERRARD A. WIGRAM, of 14 Elvaston Place, South Kensington, left £100 to the Royal Hospital for Incurables.

MISS MARY EMMA BROCK, of Holland House, Walter Road, Swansea, left £200 to Swansea Hospital.

